# Pre-Historic Teeth

**Published:** 1884-01

**Authors:** W. D. Miller

**Affiliations:** No. 2 Hausvoigtei-platz, Berlin


### PRE-HISTORIC TEETH.

No. 2 Hausvoigtei-platz,

Berlin, December 1st, 1883.

*Editor Independent Practitioner:*

Some days ago I received the October number of the Independent Practitioner, containing your communication entitled: "An examination of the condition of the teeth of certain pre-historic American races." I need not say that I was very much interested in the facts therein brought to light, more particularly because it is a subject to which I have myself given considerable attention. Not quite a year ago I examined carefully all the skulls contained in the anatomical museum of the University of Berlin, about nine hundred in number, and made a record for each case of the number of teeth present, the number of cavities of decay, their location and extent, the amount of tartar, evidences of alveolar abscess, pyorrhœa alveolaris, etc., etc. In this collection are to be found skulls from all parts of the earth and from all ages, and a study of them permits of only one conclusion, viz.: wherever man is found, there caries of the teeth and its concomitant evils will be found also.

I would not say, nor do you say, that pre-historic races were, or that modern uncivilized races are as much tormented with this disease as the modern civilized races. I should be inclined to think not; but that caries was present in its worst form, of that there can be no doubt.

There is *one* lesion of the teeth which we meet with in practice almost daily, that I have not found in my examinations,—in the whole anatomical museum there is not a single sure case of that form of erosion which is here called the "wedge-shaped defect." I have seen it somewhere stated that barbarous races also suffer from this evil; that may be so, but no such cases found their way into the Berlin museum. There are several large collections here, which I hope to be able to examine as soon as time allows, and may then give you in proper form the results of my examination.

At present let me refer to a very few cases.

1. Skull from an old burial place of the Huns, near Kertsch; sixteen teeth in position. The right superior first molar, right inferior first molar, and right inferior second bicuspid, all contained compound approximal cavities; there were traces of caries on the grinding surface of all the molars.

2. Skull from same grave; eleven teeth in position; one compound cavity, one exposure of pulp, and all the left upper molars gone, and alveolus obliterated.

3. Skull dug out of the ground in Sicily, very old, exact date uncertain; six teeth in position, all containing compound cavities on the grinding surface.

4. Mummy; thirty-two teeth; two simple and two compound buccal cavities, and one leading to exposure of pulp; right superior second molar and left superior first bicuspid complete ruins.

5. Ancient skull from a grave near Cairo; seventeen teeth. Upper molars all lost during life, except the left first, which was decayed to the margin of the gum. The left superior second bicuspid was in the same condition, both extensively abscessed, the whole external alveolar wall being gone completely, exposing the roots of both teeth.

6. Skull of a child from ancient Peru; one of the first molars a complete ruin; others had compound cavities on the grinding surface. Three of the temporary molars contained compound approximal cavities, and one was abscessed.

7. Skull of a Guarani (Indian race in Paraguay); thirty-two teeth. This denture would almost rival anything that could be shown to-day in Berlin or New York. There were no less than twenty-three large cavities, and three teeth decayed to the gum.

Of course the above are some of the worst cases, and there were all gradations from those named down to perfect dentures. There were also a number of cases where the teeth had all been lost during life, also abundant evidences of pyorrhœa alveolaris, and large accumulations of tartar. By the way, I did not fail in any case examined with reference to this point to find the familiar micro-organisms in the tartar after decalcification.

One denture, that of a Javanese, presented an appearance as "natural as life"; the left superior first bicuspid stood inside of the arch, forming an equilateral triangle with the cuspid and second bicuspid; all three teeth were extensively decayed on the surface looking toward the center of the triangle.

In looking over the tabulated results obtained from the examination of these nine hundred specimens, I am able to draw no other conclusion than this: whatever deleterious effects the vices of civilization may have had upon the structure of the teeth, however much their power to resist the agents of decay may thereby have been reduced, the origin of the disease *caries dentium* itself, must be sought for at a time and place where neither modern nor civilizing influences had any existence.

Very truly yours,

W. D. MILLER.

